# Basic psychological needs satisfaction of stroke patients: a qualitative study

**DOI:** 10.1186/s40359-023-01107-4

**Published:** 2023-03-07

**Authors:** Huiqi Lu, Xiyi Tan, Xiangmin Wang, Qinger Lin, Simin Huang, Jinjun Li, Hongzhen Zhou

**Affiliations:** 1 Department of Nursing, Nanfang Hospital, Southern Medical University, Guangzhou, China; 2 School of Nursing, Southern Medical University, Guangzhou, China

**Keywords:** Stroke, Basic psychological needs, Satisfaction, Qualitative

## Abstract

**Background:**

Previous studies have shown that the satisfaction of basic psychological needs is related to psychological well-being. Improving satisfaction will increase personal well-being, promote positive health outcomes, and improve disease recovery. However, no research has focused on the basic psychological needs of stroke patients. Therefore, this study aims to determine the basic psychological needs experience, satisfaction, and its influencing factors of stroke patients.

**Methods:**

12 males and 6 females in the non-acute phase with stroke were recruited in the Department of Neurology, Nanfang Hospital. The individual, semi-structured interviews were conducted in a separate room. The data were imported to Nvivo 12 and analyzed using the directed content analysis approach.

**Results:**

Three main themes consisting of 9 sub-themes were derived from the analysis. These three main themes focused on the needs for autonomy, competence, and relatedness of stroke patients.

**Conclusion:**

Participants have different degrees of satisfaction of their basic psychological needs, which may be related to their family environment, work environment, stroke symptoms, or other factors. Stroke symptoms can significantly reduce the patients’ needs for autonomy and competence. However, the stroke seems to increase the patients’ satisfaction of the need for relatedness.

**Supplementary Information:**

The online version contains supplementary material available at 10.1186/s40359-023-01107-4.

## Background

Stroke is the second leading cause of death and disability worldwide, especially in low- and middle-income countries [1], with the characteristics of high morbidity, mortality, disability rate, and many complications. With the highest disability-adjusted life-years (DALYs) of any other disease in China [2], the disability of stroke mainly manifests in the changes of appearance caused by facial paralysis, dyskinesia, dysphagia, speech disorder, and visual and hearing impairment. Disability and dysfunction seriously affect the ability of patients to take care of themselves and social interaction, which increases the burden on their families, and leads patients to negative emotions. Due to the psychological burden and pathophysiological factors of stroke, approximately one-third of patients suffer from depression after stroke [3]. And patients with post-stroke depression (PSD) have poor treatment adherence, prognosis, and low quality of life, compared to patients without PSD. Therefore, it’s necessary to carry out psychological interventions according to patients’ psychological burdens and needs.

Basic psychological needs theory (BPNT), developed by American psychologists Deci and Ryan, is the core theory of self-determination theory (SDT), including three parts: the need for autonomy, competence, and relatedness. Deci and Ryan point out that there are specifiable psychological and social nutrients that, when satisfied within the interpersonal and cultural contexts of an individual’s development, can facilitate one’s growth, integrity, and well-being. And they refer to these necessary satisfactions for personality and cognitive growth as basic psychological needs (BPN) [4]. Autonomy refers to feeling willingness and volition for one’s behaviors. Competence refers to feeling effective in one’s interactions with the social environment—that is, experiencing opportunities and supports for the exercise, expansion, and expression of one’s capacities and talents. Relatedness refers to both experiencing others as responsive and sensitive and being able to be responsive and sensitive to them—that is, feeling connected and involved with others and having a sense of belonging [5].

Based on empirical studies, the satisfaction of basic psychological needs will increase an individual’s well-being, promote positive health outcomes and facilitate the rehabilitation of diseases. Research on the smoker’s health project has shown that the satisfaction of BPN could facilitate long-term tobacco abstinence [6]. A diabetes management plan based on BPNT was proven effective in lowering the patient’s glucose level [7]. However, few studies focused on the BPN of stroke patients, so there is a gap in knowledge about what they need and how to satisfy their needs. Therefore, the current study aimed to determine the performance, satisfaction, and influencing factors of BPN in stroke patients.

## Methods

### Study design and setting

We conducted a qualitative study using individual, semi-structured interviews to examine participants’ perceived needs satisfaction after stroke. In the interview, we could explore the participants’ experiences with autonomy, competence, and relatedness to create a comprehensive, deep understanding of the influencing factors that are relevant to the satisfaction of BPN. The interviews were conducted in a separate room in the Department of Neurology, Nanfang Hospital, when participants were free from any treatment and therapy.

### Participants

In this study, the selection criteria of the participants were the following: (1) diagnosed with stroke, regardless of the type; (2) confirmed to be in the non-acute phase by the doctor; and (3) without speech disorder. The researcher checked the information and files of potential participants and asked if they would like to join the study.

### Ethics

This study was reviewed and approved by the Medical Ethics Committee of Nanfang Hospital of Southern Medical University. All participants signed the informed consent after receiving the purpose and procedures of the study, especially the audio and handwriting recording throughout the interview.

### Data collection

The individual, semi-structured interviews were conducted by the first author, who had been trained for the qualitative interview. Before the interview, the interviewer would create a private, respectful, and relaxing atmosphere to ensure that the participant felt cared for and would like to speak freely without any hesitation, to improve the quality of the interview. An interview guide was developed to conduct the interview based on BPNT and previous researches. It included six main questions. (1) Talk about your rehabilitation plan. Do you find it challenging for you? Are you willing to persist in completing your rehabilitation training? What supported you to persist in completing rehabilitation training? (2) Did you do any work before you got sick? (If yes) Would you consider returning to work later? What makes you feel you can/can’t continue working? (3) Talk about your hobbies and have you continued to carry out your hobbies after stroke? (4) Talk about your autonomous choices during treatment, rehabilitation, and daily life. (5) How well do you get along with the people around you after stroke? Do you feel that the way you get along with the people around you is different from before you got stroke? How do you feel about this change? (6) what do you think is the most significant impact that the stroke has on you? During the interview, we wished to explore the basic psychological needs of patients after stroke. For example, their autonomous choices in various aspects of their lives, and changes in their hobbies that were considered autonomous behaviors. And if there were any changes in the patient’s abilities in daily life, work, treatment, and rehabilitation. Finally, we wanted to talk about the relationship between patients and the people around them. These phenomena that we pay attention to also became the units of analysis in the next step of data analysis. The interviewer used a mobile phone to record the entire interview and noted some key phrases and participants’ facial expressions. Both the audio records and written notes were informed to the participants before the interview. Data collection and data analysis were carried out simultaneously. Participant recruitment was stopped when the codes were repeated and no new themes appeared in the data analysis [8]. For example, the code “competence” refers to the ability that the participant demonstrated in a particular task or job. And themes are identified as the manifestation, satisfaction, or frustration of a psychological need of the participants in a particular environment, for example, the satisfaction of the need for autonomy in daily life. In other words, when exploring the basic psychological needs of patients, no new manifestations of BPN or new needs emerged, and the research sample reached saturation.

### Data analysis

After each interview, 2 researchers (LH and TX) listened to the audio record, checked the handwritten note, and transcribed them into written transcripts within 12 h. Then 2 researchers checked their transcripts, and if any discrepancies were found, they would listen to the audio record again until they reached a consensus. All transcripts were imported to Nvivo 12, a software used to analyze qualitative data. Two researchers (WX and LQ), who had an experience in qualitative research and learned about BPNT, participated in the data analysis. The directed content analysis approach was used to analyze data, which is appropriate where an existing theory can guide research questions and initial theory-based themes and can be supported, challenged, or extended [9]. This analysis approach has the following steps. (1) Identify key concepts as initial coding framework based on existing theory and published researches. (2) Carefully read and record all transcripts in detail and encode the them; (3) Induce and abstract all codes, and classify them according to the initial coding framework; (4) Codes that cannot be classified using the initial coding framework will be given a new theme. In this study, WX and LQ started with the first three transcripts. They read the transcripts carefully, discussed the initial codes, and determined the initial coding framework based on the BPNT. That is, the need for autonomy, the need for competence, and the need for relatedness in BPNT were identified as three main themes in the analysis. The units of analysis have been described above, and among them, the three basic psychological needs were the most basic and critical units of analysis. All transcripts were divided equally to two researchers to be analyzed and coded. All codes were incorporated into three main themes in the initial coding framework, and new main themes can be determined if needed. And the codes were induced and abstracted into themes. As the coding level progressed, three levels of themes emerged and their representative quotes were determined. Challenges and disagreements in the structures and meanings associated with the codebook, were resolved through consensus in regular group meetings including all co-authors. Leading to the final directed content analysis presented in the results.

## Results

Eighteen participants participated in the semi-structured interviews, including 12 males (66.67%) and 6 females (33.33%) (Table 1). The mean age of participants was 57.56 ± 10.67 years. Almost all participants (88.89%) were diagnosed with ischemic stroke. And they had a median course of stroke of 5 months (range 0.5 to 48 months). Only 5 participants (27.78%) were still working after stroke. And almost all participants (94.44%) lived with their family. The median time duration of the interviews is 14 min (rage 10 to 55 min). All participants were named with English letters as their code names to protect their privacy.

**Table 1:** Participant characteristics

Participant	Age (years)	Gender	Course of stroke (months)	Type of stroke	Occupation	Residential status
A	45	Male	5.0	a	Working	With family
B	54	Male	1.0	a	Not working	With family
C	80	Male	1.0	a	Not working	With family
D	62	Female	6.0	a	Working	Alone
E	68	Female	18.0	b	Not working	With family
F	43	Female	0.5	a	Not working	With family
G	59	Male	1.0	a	Not working	With family
H	64	Female	1.0	a	Working	With family
I	58	Female	9.0	a	Working	With family
J	65	Male	11.0	a	Not working	With family
K	34	Male	10.0	a	Not working	With family
L	65	Male	48.0	a	Not working	With family
M	48	Male	10.0	a	Not working	With family
N	57	Male	4.0	a	Working	With family
O	55	Female	5.0	a	Not working	With family
P	61	Male	10.0	b	Not working	With family
Q	52	Male	2.0	a	Not working	With family
R	66	Male	5.0	a	Not working	With family

a refers to ‘Cerebral ischemia’, b refers to ‘Intracerebral hemorrhage’.

Based on basic psychological needs theory, participants talked about their needs for autonomy, competence, and relatedness during treatment and rehabilitation after stroke. According to the interview guide, the information in Question 1 and 2 are particularly associated with the need for competence. And the information in Question 3 presents 3 basic psychological needs of stroke patients. The information obtained from question 4 mainly describes the patients’ needs for autonomy. And the information in Question 5 focuses primarily on their needs for relatedness. As an open question, the information obtained from question 6 is considered to be associated with 3 basic psychological needs. As components of BPN, these three needs were identified as the main themes, according to directed content analysis. And data analysis yielded 115 codes dispersed in 31 sub-subthemes belonging to 9 sub-themes (Table 2). There were 10 sub-subthemes in main theme 1, 13 in main theme 2, and 8 in main theme 3. Since each sub-subtheme contains fewer quotes and implications, the Results and Discussion sections would mainly revolve around the sub-themes with more quotes and implications. All themes and their corresponding quotes are presented in the appendix. The result is presented with their quotes along with code name, gender, age, and course of stroke.

**Table 2:** Main themes and sub-themes

Main theme	Sub-theme	number of narrators (%)
the need for autonomy	the need for autonomy in daily life	11 (61.11)
	the need for autonomy during treatment and rehabilitation	11 (61.11)
	making lifestyle changes consciously	10 (55.56)
the need for competence	the impact of stroke on the ability of daily living activities	11 (61.11)
	the impact of stroke on the ability to work	10 (55.56)
	the need for competence during treatment and rehabilitation	11 (61.11)
the need for relatedness	the need for relatedness during hospitalization	7 (38.89)
	the need for relatedness in the rehabilitation stage	9 (50.00)
	the impact of stroke on relationships	6 (33.33)

### Main theme 1: the need for autonomy

The participants’ need for autonomy is reflected in various aspects, such as work, medical treatment, and activities in daily life. Although they have a mean age of 57.56 years, and most have retired or left the working environment, they still have tasks that can be called work in their daily life, like, doing housework and raising grandchildren. After abstracting and summarizing, this main theme includes three sub-themes, (1) the need for autonomy in daily life, (2) the need for autonomy during treatment and rehabilitation, and (3) making lifestyle changes consciously. There are 10 sub-subthemes included which are described in each sub-theme.

### Sub-theme 1.1: the need for autonomy in daily life

#### Sub-subthemes 1.1.1 at a high degree of autonomy satisfaction in daily life

Most participants said that there were few things they had to do in daily life, and most of the things they did were out of their own will, that is to say, that participants were at a high degree of autonomy need satisfaction in daily life.

*When it comes to food, my son said: “Mom, you can buy whatever you want. It’s not that we don’t have money, so don’t worry about it.” … And I can do what I want to do. Even though my son told me not to (do something), as long as I want to or like to, then I can do it in the end. (H, female, 64, a month after stroke diagnosis)*

#### Sub-subthemes 1.1.2 an increased sense of autonomy in housework after stroke

After the stroke, most things changed in the participants’ life. A participant who had done housework for many years felt so tired of the overwhelming housework before stroke. And after stroke, she felt an increased sense of autonomy in housework, which made her feel relaxed and happy.

*Before I got sick, I had to do housework even if I didn’t want to. When I saw things dirty and thrown everywhere, I could do nothing but clean them up…However, it comes different now. They do the housework, and I could do something if I want to and vice versa. (D, female, 62, 6 months after stroke diagnosis)*

#### Sub-subthemes 1.1.3 do not feeling free during hospitalization

However, the disease also has adverse effects on the participants’ need for autonomy. Another participant felt sad about this and was reluctant to talk more about autonomy.

*This… (a long silence) is that I don’t feel free and can’t do many things I want to do. (Researcher: “what do you want to do?“ The participant looked left and right repeatedly and did not answer the question.) (G, male, 59, a month after stroke diagnosis)*.

#### Sub-subthemes 1.1.4 the impact of stroke on interests

When it comes to autonomy, interest is seen as a primitive form of intrinsic motivation, which promotes the satisfaction of the need for autonomy. Different people have different interests. Participants who enjoy high-intensity activities such as running and dancing said they didn’t dare to continue doing these. But as for reading books or watching TY, stroke has little impact on these activities.

*I usually just play TikTok and watch TV. Because I feel dizzy sometimes after stroke, I don’t dare to dance, which I used to. (E, female, 68, 18 months after stroke diagnosis)*

### Sub-theme 1.2: the need for autonomy during treatment and rehabilitation

#### Sub-subthemes 1.2.1 the sense of autonomy during treatment and rehabilitation

During treatment and rehabilitation, participants also have a sense of autonomy in making medical decisions. Especially in physiotherapy and rehabilitation training, they have more choices, which satisfies the need for autonomy.

*Just like doing this (medium frequency physiotherapy), I’ll tell you where I feel uncomfortable and where I decide to do it. (D, female, 62, 6 months after stroke diagnosis)*

#### Sub-subthemes 1.2.2 willing to follow the advice of a medical professional

However, it needs to be clear that the term autonomy does not refer to independence. People can do something independently and act volitionally. Yet, people can also depend on others as they would like to, which represents autonomous dependence. It means that participants willingly chose to follow medical professionals’ advice, which is also a manifestation of a sense of autonomy.

*I have a good friend who is a doctor and a professor. I would always listen to his opinions. For example, I have been taking this medicine for a long time, which he introduced me to take. (C, male, 80, a month after stroke diagnosis)*

#### Sub-subthemes 1.2.3 less self-determination due to the lack of knowledge

Unfortunately, there are few choices for patients to make in the medical decision. Perhaps one of the reasons is that they lack enough knowledge to make autonomous decisions.

*I have been in the hospital for many months, and now I just want to go home, but I am not sure about that. Because I need to do rehabilitation training, staying in the hospital will have a better effect. However, I would feel better at home so that I may recover well. Therefore, I don’t know how to make a decision. (R, male, 66, 5 months after stroke diagnosis)*

### Sub-theme 1.3: making lifestyle changes consciously

In the interviews, we found an interesting phenomenon that almost all participants mentioned that they did think that they had engaging in some behaviors that are known as risk factors for stroke. Not only that, but some also said that they would change or had changed these unhealthy lifestyles consciously.

#### Sub-subthemes 1.3.1 correctness of unhealthy behavior in daily life

A participant who had drunk and smoked for many years said that, although many people told him it was not good for his health in these years, he never thought about making changes until this stroke.

*I have been smoking and drinking since I was 20 years old. Now I would not smoke and drink anymore since I got stroke. I always told them to stop showing me alcohol and cigarettes. (G, male, 59, a month after stroke diagnosis)*

One participant noted that her personality might have something to do with her stroke.

*I feel like I’m being too impatient. I would get everything done at once and not stop until it was finished. (D, female, 62, 6 months)*

#### Sub-subthemes 1.3.2 correctness of unhealthy eating habits

Besides the unhealthy behavior in daily life, many participants had unhealthy eating habits, such as high-fat and high-salt diets.

*I eat less than before, especially rice, but more vegetables. And I am on a low-fat and low-salt diet. (P, male, 61, 10 months after stroke diagnosis)*

#### Sub-subthemes 1.3.3 development of an exercising habit

Due to the rehabilitation training, some participants developed a habit of exercise. Some would insist on doing rehabilitation training learned from the hospital, and some would go for general sports like walking and running.

*I emphasize exercising now…Every day I would go for a walk after dinner and do some exercises or something else. (L, male, 65, 48 months after stroke diagnosis)*

### Main theme 2: the need for competence

This theme focused on the impact of stroke on participants’ ability and the need for competence. The stroke can negatively affect the patient’s physical function and impair the patient’s ability to perform various activities, which reduces the satisfaction of the need for competence. This main theme includes three sub-themes, (1) the impact of stroke on the ability of daily living activities, (2) the impact of stroke on the ability to work, and (3) the need for competence during treatment and rehabilitation. There are 13 sub-subthemes included which are described in each sub-theme.

### Sub-theme 2.1: the impact of stroke on the ability of daily living activities

#### Sub-subthemes 2.1.1 difficulty in performing some daily activities

Most participants said that stroke did produce physical symptoms that could not be ignored and impaired their daily activities, such as dizziness and numbness of limbs. Therefore, they had difficulty in performing some daily activities.

*I used to drive, but now I don’t dare. I tried for one time, but I felt that my reaction was not so responsive, such as when turning and decelerating. (M, male, 48, 10 months after stroke diagnosis)*

#### Sub-subthemes 2.1.2 difficulty in doing housework

Participants even had difficulty in doing housework due to the reduced physical mobility.

*I didn’t do any heavy work either, just do some sanitation at home… My left hand is not so neat, but my right hand can work. (O, female, 55, 5 months)*

#### Sub-subthemes 2.1.3 difficulty in caring for the family

As mentioned above, some participants usually did housework and took care of family at home, however, it was difficult for them to continue after stroke.

*I have been caring and cooking for my son because he is not married yet and no one can do this, which is very tiring. Now it’s such a hassle since I got sick, I can’t take care of him anymore. (C, male, 80, a month after stroke diagnosis)*

#### Sub-subthemes 2.1.4 part of the work is undertaken by others

Even so, some participants still continued doing the same things they did before got stroke while recovering at home. Certainly, their family members would help or undertake part of the work.

*I used to grow some vegetables at home. But now I cannot do much hard work, so my husband would do most of the work and I just have to do the easy part. (I, female, 58, 9 months after stroke diagnosis)*

#### Sub-subthemes 2.1.5 recuperating at home and no need to work

One participant said she didn’t need to do anything at home, even though it had been a long time since she was diagnosed with stroke and she was capable to do something. Thanks to her family, she is indeed felt relieved from the hectic housework finally.

*I used to do housework at home and take care of my grandchildren… And I stopped working since the cerebral hemorrhage last year. (E, female, 68, 18 months after stroke diagnosis)*

### Sub-theme 2.2: the impact of stroke on the ability to work

This sub-theme focused on work, or job, which is paid and in a specific environment. Because the medical treatment and rehabilitation would last a long time, most participants quit their jobs or asked for some days off.

#### Sub-subthemes 2.2.1 return to work

Coincidentally, two participants owned a store and worked there. They all said they would go back to work in the store after being discharged from the hospital. One participant was confident and thought she was capable of continuing to work.

*I will definitely return to work after I am discharged from the hospital. Because this disease does not affect me, I don’t need to do heavy work in the store. Ah, I work there because it’s better to have someone in the store to supervise the employees. It’s just that. It’s nothing. (H, female, 64, a month after stroke diagnosis)*

#### Sub-subthemes 2.2.2 reducing intensity and content of the work

However, the other participant said she would reduce the intensity and content of the work due to her physical condition after stroke.

*…I can only walk around, do what I can, and instruct employees to do what I can’t do. Because of dizziness, I dare not walk for too long. Maybe I should just sit there and look at the monitoring system. (D, female, 62, 6 months after stroke diagnosis)*

#### Sub-subthemes 2.2.3 planning to do something new after being discharged

Due to retirement age, one participant planned to retire and do something new after being discharged from the hospital.

*…Now I plan to renovate the house, um, the house I am living in. (G, male, 59, a month after stroke diagnosis)*

#### Sub-subthemes 2.2.4 not able to return to work yet

However, some participants failed to return to work, even though they had been in rehabilitation for quite some time.

*I don’t know how to talk about it. Anyway, I haven’t gone to work. And I just do housework and take care of my children at home. (K, male, 34, 10 months after stroke diagnosis)*

### Sub-theme 2.3: the need for competence during treatment and rehabilitation

Stroke can be a considerable challenge and change many things in participants’ life. Changes in participants’ abilities, environment, and what they need to do will affect the satisfaction of the need for competence.

#### Sub-subthemes 2.3.1 knowledge of treatment and rehabilitation

In sub-theme 1.2, it was found that lack of knowledge would impair the satisfaction of the need for autonomy, and so did it in this theme. Inadequate knowledge reduces their ability to deal with events and circumstances, making them appear somewhat helpless and have no idea what to do and how to do it during treatment and rehabilitation [10]. Some participants didn’t know how to adjust their daily diet and whether they should take medicines for a long time. And they didn’t know where and how to do rehabilitation training.

*I don’t know. (laughs) I don’t understand those things. …(hesitation) Then where can I go to do the rehabilitation training? …Oh… Is the community hospital where can do it? OK, let me ask. I often feel pain, and it’s not good, so I’ll ask the workers there. (I, female, 58, 9 months)*

Some had a misunderstanding about rehabilitation, which may slow down the recovery process.

*I guess that the effect of rehabilitation training is not significant, and I feel much better now, so I didn’t ask for the training. (Q, male, 52, 2 months after stroke diagnosis)*

Fortunately, part of the participants could obtain relevant knowledge from the Internet.

*Just when I was sleeping, I felt numb in my feet, which I had never met before. But I know that, um, sometimes I see videos in TikTok saying that because of high blood pressure, the numbness in my feet may imply a stroke. (H, female, 64, a month after stroke diagnosis)*

#### Sub-subthemes 2.3.2 rehabilitation training is within the ability

Most participants indicated that the rehabilitation training was within their ability, so they could keep doing it.

*The rehabilitation training is not difficult, and I can do it. (K, male, 34, 10 months after stroke diagnosis)*

#### Sub-subthemes 2.3.3 creating his own rehabilitation plan

And a participant was so proud to tell the researcher that he had created his own rehabilitation training plan.

*Now I do rehabilitation exercises several times a day, every day, which was designed by ourselves. (laughs) (N, male, 57, 4 months after stroke diagnosis)*.

#### Sub-subthemes 2.3.4 rehabilitation training ended for various reasons

However, some participants ended their rehabilitation for various reasons. The main reason was that they did not get an obvious effect in rehabilitation training. He may have no idea that rehabilitation training needs to last for a long time to get obvious results.

*I had been doing rehabilitation training for about half a year. Because I felt that the effect was not obvious, I stopped doing it. (E, female, 68, 18 months after stroke diagnosis)*

The other reason was that their poor physical condition resulted in poor mobility does not allow further training.

*Running sometimes makes me feel comfortable, um, sometimes it’s not. Because I have arthritis, sometimes running makes me feel painful, so I stop it. (I, female, 58, 9 months after stroke diagnosis)*

### Main theme 3: the need for relatedness

The theme focused on the impact of stroke on participants’ relationships and their need for relatedness during treatment and rehabilitation. Stroke might not only change the relationship with family and friends, but it could also develop new relationships between participants, medical workers, and wardmates. This main theme includes three sub-themes, (1) the need for relatedness during hospitalization, (2) the need for relatedness in the rehabilitation stage, and (3) the impact of stroke on relationships. There are 8 sub-subthemes included which are described in each sub-theme.

### Sub-theme 3.1: the need for relatedness during hospitalization

#### Sub-subthemes 3.1.1 longing for the company of family during hospitalization

Almost all of the participants indicated they were very eager for the company of their family during hospitalization.

*My sons and daughters-in-law all have busy jobs, so they don’t have time to come here and take care of me. And I don’t think I need someone to care for. However, sometimes when they call me, tears run down my face. (Laughs) Actually, I want someone to be here with me. (E, female, 68, 18 months after stroke diagnosis)*

In addition, a participant said that he felt happy when his family visited him and believed that being with his family would help him recover.

*I’ve recovered well now, and so do my language function. Since I got sick, I have had difficulty in speaking. For example, sometimes I didn’t know how to say something. When my grandchildren came to visit me, I felt so happy and could call their names and talk to them about anything. But after that, I couldn’t speak well again. (R, male, 66, 5 months after stroke diagnosis)*

#### Sub-subthemes 3.1.2 relatives and friends were unable to visit the patient in hospital

Although it may be difficult for relatives and friends to visit patients in hospitals, they often contact patients by phone or WeChat.

*Last night my worker sent a WeChat message asking how I was in the hospital. I said it was all good. (Laughs)… My son often calls me, asks if the food is good, and tells his wife to buy some delicious food for me. (H, female, 64, a month after stroke diagnosis)*

However, some participants chose not to inform relatives and friends of the illness, so as not to increase their burden.

*I haven’t officially told my friends about my illness, but I will definitely tell them after I am discharged…And I also wanted to tell my wife after I was cured and discharged, because I was afraid that if I told her, it would make her feel stressed and sad. (C, male, 80, a month after stroke diagnosis)*

#### Sub-subthemes 3.1.3 geting along well with healthcare workers and other patients

Although participants didn’t get enough company from family and friends, they still had healthcare workers and other patients with whom they got along well. Most participants felt that they got enough care from healthcare workers.

*I have a lot of confidence in you all. You are all professional and care much about (me), so I trust you. In a word, you doctors and nurses are all very responsible in all aspects. (A, male, 45, 5 months after stroke diagnosis)*

But he didn’t talk too much with other patients.

*When it comes to other patients in the ward, I don’t talk to them too much. Because I don’t know exactly how they are, I dare not laugh and act too happy if they feel so bad. (A, male, 45, 5 months after stroke diagnosis)*

### Sub-theme 3.2: the need for relatedness in the rehabilitation stage

#### Sub-subthemes 3.2.1 rehabilitation training accompanied by family members

After being discharged from the hospital, participants moved into a rehabilitation stage. Most participants stay at home or go to specific institutions for rehabilitation training at this stage. And the training takes a long time. Therefore, most family members would accompany the participants to do the exercise or training.

*I do rehabilitation exercises every day, (laughs), and my wife also exercises with me at least three times a day. After the exercises, she also massages my hands and feet. (N, male, 57, 4 months after stroke diagnosis)*

#### Sub-subthemes 3.2.2 receiving help from other patients

Those who go to specific institutions for rehabilitation training can often meet other patients and get their help.

*On the past day, the older adults in the rehabilitation institution said that the training was helpful, so I went there and tried. And I also bought a physiotherapy device they recommended, and I use it every day. (E, female, 68, 18 months after stroke diagnosis)*

#### Sub-subthemes 3.2.3 socializing with friends

As participants’ physical function recovered, their lives went back on track. They usually socialized with friends in their spare time, such as playing cards, traveling, etc.

*I live in the countryside and don’t have much to do. I usually play cards and chat with the people next door, which makes me quite happy. (O, female, 55, 5 months after stroke diagnosis)*

### Sub-theme 3.3: impact of stroke on relationships

#### Sub-subthemes 3.3.1 feeling like family cares more about him

Through interviews, almost all the participants said their relationships with their families improved after stroke. Because of the illness, they felt that family cared much more about them than before, and so did friends.

*The way that my family treats me must be different. For example, the time when they come home has been changed. In the past, when they came back, I had already been asleep…And nowadays, when I felt a little bit not good, they became so nervous and wanted to send me to the hospital. (N, male, 57, 4 months after stroke diagnosis)*

#### Sub-subthemes 3.3.2 the response of company staff to the illness

However, the relationship between participants, and their workmates or boss is much more complicated. The response of company staff to his illness can be very different. A participant said he had a considerate boss, so he successfully got sick leave.

*My boss was so concerned about me and he just gave me a sick leave. And he told me not to return to work until I recovered. (A, male, 45, 5 months after stroke diagnosis)*

But unfortunately, another participant lost his job. He appeared a little sad and angry.

*I worked before but would not return to work after being discharged. They said I didn’t need to go back, and how do I know why! Definitely, they thought I needed to have a long break! (B, male, 54, a month after stroke diagnosis)*

## Discussion

This study was the first qualitative study that examined the basic psychological need of stroke patients and analyzed the influencing factors and satisfaction of the needs. Based on BPNT, the initial coding framework was determined to be 3 themes that were, (1) the need for autonomy, (2) the need for competence, and (3) the need for relatedness. And this framework was also validated by the analysis result, because no code could not be classified into these three themes. Below, we reflect on each of these main themes.

### The need for autonomy

#### The need for autonomy in daily life

The majority of stroke patients are older adults. And in this study, 44.44% of the participants are older than 60. Most of them did not participate in work or had retired, so they had much leisure time in their daily life to do what they liked. In addition, they had stable and sufficient financial resources, such as pensions. Therefore, when being asked, most of the participants said without hesitation that they could do what they wanted and not do what they didn’t want to do in daily life, which means that they had a high satisfaction degree of the need for autonomy in their daily life [11]. Of course, there are exceptions. In most Chinese families, due to being idle at home, the older adults need to undertake the tasks of doing housework and taking care of grandchildren to reduce the burden on their children [12], which may also be a heavy burden and occupying almost all the time of the older adults, weakening their sense of autonomy. It was found in the interviews that the sense of autonomy has a greater relationship with their home environment and relationship with their families. They get more negative emotions when they are overburdened with tasks and don’t get any understanding or help from their families, which may usually lead to depression [13]. However, things changed after stroke. Due to recuperating from the disease, the patients are relieved of all the things they had to do in the past and have more time to make their own arrangements. Therefore, they have a higher satisfaction degree of the need for autonomy after stroke. But beyond that, the disease may impair patients’ physical functions, reducing their sense of autonomy [14]. Hobbies are purely enjoyable activities that manifest a sense of autonomy and can also be affected by the disease [4]. For example, patients can’t run or dance because of limb numbness or decreased muscle tone, and they can’t watch TV or read a book due to dizziness or impaired visual function. Especially during hospitalization, patients can feel trapped and lose freedom. Decreased satisfaction of the need for autonomy can cause negative emotions, which are detrimental to mental health and recovery from stroke [15].

#### The need for autonomy during treatment and rehabilitation

Regarding treatment and rehabilitation, especially during hospitalization, obedience and dependence are the first things that come to mind, instead of autonomy. It is true that the main things for stroke patients to do during hospitalization are to follow the doctor’s instructions for infusion, taking medicine, doing examinations, and doing rehabilitation. It seems that there is no room for patients to make their own decisions. However, as we have emphasized, autonomy does not equal independence [4]. There may be a patient whose self-care ability has decreased due to a stroke and needs the care of others temporarily. If the caregiver is a close and trusted person to this patient, he can rely on the caregiver, which is also a manifestation of autonomy [16]. Most participants said that all they had to do in the hospital was listen to the doctor and follow the treatment plan, because healthcare workers were professional in helping patients recover from the disease. At the same time, hospitals are paying more attention to shared decision-making [17]. When there are different treatment plans, doctors will invite patients and their families to participate in medical decision-making, which can meet the needs of patients’ autonomy [18]. The essential thing in shared decision-making is that the details, advantages, and disadvantages of different treatment plans should be correctly and unreservedly informed to patients, so that they have a comprehensive understanding of the plans, which helps them make a decision.

### Making lifestyle changes consciously

As we all know, it is so difficult to change a habit or a lifestyle. A study showed that initiating and maintaining lifestyle changes is a long and complex process [19]. It is not so difficult to initiate the lifestyle change, and controlled motivations can play a role in the initiation. For example, patients are told that this lifestyle is unhealthy and needs to be changed. On the contrary, maintenance of lifestyle changes is the harder and more critical part [20]. In this stage, controlled motivation is not enough, and autonomous motivation is required. In other words, lifestyle changes need patients to have a greater sense of autonomy. It was so nice to see that in the interviews, most of the participants shortly after the onset of stroke were able to understand and accept that they needed to change their unhealthy lifestyles and confidently reassure the researchers that they would change in the future. Some participants with a longer course of disease said they had successfully changed their previous unhealthy lifestyles and could consciously continue to maintain them. And what can we do to help them initiate and maintain lifestyle changes? The first thing is to provide correct, detailed, and accessible health education to patients, so that they realize that they have an unhealthy lifestyle [21]. For example, hospitals and communities regularly conduct health classes, produce brochures, and disseminate health knowledge through the Internet. And the most important is how to help them maintain their lifestyle changes. Web applications are widely used today and can also be used for that. For example, patients can set a goal in advance, then self-check or be checked every day to determine whether the goal has been met. Rewards can also be incorporated into the application to provide some motivation. An experiment showed that the application offered instructions for a healthy lifestyle effectively promoted a lifestyle change [22].

### The need for competence

#### The impact of stroke on the ability of daily living activities

Unlike the need for autonomy, the need for competence is undermined by stroke. Patients experience different degrees of functional impairment after stroke. According to surveys, about 70% of patients have dysfunction and disability, which seriously affects their activities in daily life, including Activities of Daily Living (ADLs) and Instrumental Activities of Daily Living (IADLs) [23]. ADLs refers to daily household-based activities such as eating, going to the toilet, and dressing. IADLs refers to driving and taking transportation. If these self-care activities, which are regarded as easy things, cannot be completed, the patient may feel that he has lost control of his own life, which may lead to negative emotions, that may be closely related to post-stroke depression [3]. rehabilitation medicine is booming now, and different types of rehabilitation training are provided to patients with various functional disorders. Occupational therapy, for example, aims to improve the patient’s ability to perform activities of daily living, so that the patient can complete self-care activities independently without relying on others [24]. Similar to autonomous dependence mentioned above, is there independent in-competence? That is, whether the patients would choose to demonstrate ineffective interactions with the environment. However, this study gave a negative answer. Unlike being unable to take care of themselves, patients seem to be a little anxious and guilty when they can no longer take care of their family members. Influenced by the unique traditional culture, most families in China are stem families. That is, parents live with a pair of married children and usually grandchildren [25]. In such families, the older adults are usually responsible for housework and caring for the family, especially grandchildren. They are more inclined to make contributions to the family in order to reduce the burden on their family members, which is contrary to “independent in-competence”. Under such circumstances, patients are transformed from caregivers to care recipients, and their work is taken on by family members, which reduces the sense of competence they feel in life and impair the satisfaction of need for competence.

#### The impact of stroke on the ability to work

A study showed that the satisfaction of the need for competence in the older adults has the lowest contribution to basic psychological needs [26], which may be related to the fact that the older adults do not have many activities in their daily lives that demonstrate capacity and talent. For young stroke patients who are still engaged in work, the stroke significantly affects their need for competence. Almost all patients suspend work or even quit their jobs after stroke. And the time to return to work varies, mainly depending on the patient’s recovery and prognosis [27]. Although most young patients recover quickly from stroke symptoms, other functional impairments, such as cognitive impairment, may persist and impede return to work [28]. And completing the work tasks is the best to meet the need for competence. Most patients look forward to returning to work rather than doing nothing at home. They will think that if they are not involved in work and idle at home, they will look useless and unhelpful. Therefore, many patients said they knew their work ability and energy were not as good as before the stroke, but they still hoped to return to work, even if the work intensity and content were reduced. Moreover, returning to work also appears to enhance patients’ self-perceived participation and autonomy [29]. As a result, it is important to provide work-oriented information and rehabilitation support for working-age patients.

#### The need for competence during treatment and rehabilitation

As mentioned above, the stroke changes the patient’s life, and their daily activities and work are forced to stop. However, stroke also brings new challenges for patients, that is, insisting on rehabilitation training and learning disease-related knowledge to help recovery and prevent a recurrence. Patients with functional impairment may be hospitalized in a specific rehabilitation hospital, go to a rehabilitation institution every day or a few days a week, or perform rehabilitation training at home according to the doctor’s guidance [30]. Either option can be challenging for patients. Nonetheless, patients indicated that rehabilitation training was within their ability, regardless of the rehabilitation method chosen. Even a patient who did exercises at home proudly said that she designed the rehabilitation training program by herself, and could insist on completing it every day, which made her experience a strong sense of competence and greatly satisfied her need for competence. However, not all patients can adhere to rehabilitation training. The financial burden and poor physical condition force patients to stop rehabilitation training [31, 32], frustrating the rehabilitation training is useless or not so effective, so they stop training [32]. When it comes to disease-related knowledge, this is a more complex issue. Thanks to the Internet, patients can easily obtain a variety of information, allowing them to have a certain degree of understanding and mastery of their physical conditions, and they will feel that they are effectively dealing with disease-related matters. However, not all information on the Internet is correct, and it is difficult for patients to judge the quality of online information [33, 34]. Likewise, hospitals often produce brochures, web articles, and videos to educate patients about stroke, medication, recovery, etc. [35] But it is difficult to guarantee that patients can actually acquire knowledge from it. Therefore, it is essential to create an environment where patients can easily acquire the correct knowledge, improving their disease-related knowledge and satisfying their need for competence.

### The need for relatedness

#### The need for relatedness during hospitalization

It’s a whole new environment for a patient to be hospitalized after stroke. And according to the coronavirus disease 2019 (COVID-19) protection policy, almost all hospitals only allow patients to have one chaperone in the hospital. Having only one familiar person in a new environment can be a threat to the satisfaction of the patient’s need for relatedness [36]. To make matters worse, for various reasons, some patients are hospitalized alone, without anyone to accompany them. Feelings of loneliness and lack of support make patients feel sad or other negative emotions, which may be detrimental to their treatment and recovery from the stroke [37]. Especially when patients need to go to different wards for examination, the company of family instead of the hospital staff will make them feel more secure. In addition, the company of family may allow patients to quickly adapt to the new environment of the hospital and gain a sense of belonging. Studies have shown that family companionship, especially long-term companionship, improves patients’ functional impairment and promotes recovery [38]. Due to the widespread use of smartphones, even if relatives and friends cannot visit and accompany patients in the hospital, they will frequently contact patients to express their concerns. Even though they may be far apart in space, they will feel connected in spirit and satisfy patients’ need for relatedness. The good news is that most of the patients get along well with healthcare workers and get enough care from the workers, especially the nurses who get along day and night. A good doctor-patient relationship helps to promote treatment progress and improve their physical and mental condition [39].

#### The need for relatedness in the rehabilitation stage

During the rehabilitation stage, in addition to the patients who remain in the hospital, patients can reach more people and get support and help from them. For patients who recuperate at home, their family members will accompany them to do rehabilitation training, such as exercises, walking, and massage, making them feel closer to their families [40]. Meanwhile, most patients reported that their families gave more attention to their physical and mental condition to find the problem and provide help at the first time. Patients in a rehabilitation institution can meet fellow patients with the same condition. They usually communicate about the disease, medication, rehabilitation training, etc., from which they exchange experiences and help each other, which is very important for a chronic illness with a long recovery period such as stroke [41]. In conclusion, during the rehabilitation stage, patients may form new relationships and strengthen relationships with family members, especially caregivers, both of which satisfy the need for competence.

#### The impact of stroke on relationships

As mentioned above, the stroke strengthens the patient’s connection with his family and friends and gives the patient an opportunity to form new relationships, which greatly satisfies the patient’s need for relatedness. Another point that cannot be ignored is that the reaction and attitude of colleagues and bosses of patients who participated in work before stroke can have a great impact. If the patient is cared for, understood, and given enough vacation time for treatment and rehabilitation, he can devote himself to treatment and rehabilitation wholeheartedly and without burden [42]. And the patient can have a good expectation of returning to work after his physical condition recoveries, which may improve the patient’s physical and mental health. However, if the patient only gets complaints or even dismissal, that certainly has a negative impact on the patient’s recovery.

### Strengths and limitations

Although more and more scholars pay attention to the basic psychological needs of patients, there is still no research focusing on the satisfaction of BPN of stroke patients. This study is the first qualitative study to explore their psychological experience, satisfaction, and influencing factors in stroke patients. The qualitative data provide a rich first-person resource that lays the foundation for subsequent quantitative research of BPN in stroke patients. Based on the results of the qualitative study, we verified that BPNT is also applicable to stroke patients. Therefore, future research can focus on using BPN scales to determine the satisfaction of patients’ BPN, and to make interventions for them. When recruiting subjects, we followed the principle of information saturation and included 18 participants. And based on the widely applied theory of BPN, this study aimed to explore the BPN of stroke patients. Moreover, the researchers have been trained for qualitative interviews, and most of the participants can clearly answer the questions in the interview guide. Therefore, the narrow study aim, the applied established theory and the strong quality of dialogue indicate a high information power in this study [43]. In addition, we followed the principle of maximum variation [9] and thoroughly considered the variety of participants’ age, gender, occupation, and disease course, which enhanced the credibility and representativeness of the results. However, limited by the clinical status of the patients, only a small number of patients with intracerebral hemorrhage were recruited. The quotes were fully displayed in the results section, and all the results and their corresponding quotes were presented in an additional table [see Supplementary Information], which improved the reliability of the results [44]. Although the researchers have systematically learned qualitative interviews and content analysis methods, they are all members of the same research group and not from multiple backgrounds, which may have greater limitations in data analysis. As Hsieh said, there are certain limitations in the directed content analysis approach based on existing theory [9]. Researchers may analyze data so biased that they are hard to find evidence that doesn’t support the theory. When determining the research plan, our researchers did consider this issue and set a more open question in the interview guide, that is, question 6, to explore whether stroke patients have psychological needs beyond BPN. However, all codes could be classified into the initial coding framework, and no unclassifiable codes were generated in the data analysis. On the one hand, this result may verify the foundation and universality of BPNT. The psychological needs of stroke patients can be described by three basic psychological needs. On the other hand, perhaps researchers are so immersed in the theory that during the process of data collection and analysis, it is easier to find evidence for the theory than evidence against it.

A number of post-stroke patients lives with a speech or language disorder. However, limited by interviews, this study excluded these patients with speech disorder, which is also a limitation of this study. Obviously, speech disorder will greatly affect the satisfaction of basic psychological needs of patients. Difficulty in expressing one’s own opinions can impair his autonomous decisions and reduce his sense of autonomy [45]. At the same time, speech disorder can delay a patient’s return to work, frustrating his need for competence. The impact of speech disorder on the need for relatedness can be complex. As this study found, patients during the rehabilitation stage have more time to spend with family and friends, as well as the opportunities to form new relationships with healthcare workers and other patients. Due to speech disorder, patients need more help from caregivers, get more care from them, which may promote the satisfaction of the need for relatedness. However, due to difficulty in expressing themselves accurately, patients may be misunderstood by others, resulting in disagreements and conflicts, which may frustrate the need for relatedness. Therefore, more research is needed to focus on the basic psychological needs of patients with speech impairment after stroke.

## Conclusion

Improving the satisfaction of patients’ basic psychological needs can improve the patient’s physical and psychological status and promote the recovery and prognosis of the disease. In this study, it was found that the impact of stroke on the satisfaction of patients’ BPN is various, and the satisfaction of patients also has a large discrepancy, which may be related to their family environment, work environment, stroke symptoms, or other factors. In particular, a symptom like limb numbness can significantly reduce the satisfaction of patients’ needs for autonomy and competence. We recommend developing individualized rehabilitation training for patients to facilitate recovery and return to work. However, the stroke seems to increase the patients’ satisfaction of the need for relatedness. Hospitals and healthcare workers can improve patient satisfaction through many measures, such as listening to patients’ opinions and doing health education to improve patients’ knowledge. The hospital can create a warm atmosphere, improve the doctor-patient relationship, and enhance the patient’s sense of belonging during hospitalization. After discharge, we encourage the family to accompany patients through rehabilitation training. And patients are encouraged to go out to meet new people or socialize with friends.

## Electronic supplementary material

Below is the link to the electronic supplementary material.

Supplementary Material 1

## Data Availability

The datasets used during the study are available from the corresponding author by reasonable request.

## Abbreviations

DALYs: Disability-adjusted life-years
PSD: Post-stroke depression
BPNT: Basic psychological needs theory
SDT: Self-determination theory
BPN: Basic psychological needs
ADLs: Activities of Daily Living
IADLs: Instrumental Activities of Daily Living
COVID-19: Coronavirus disease 2019
